# Accuracy of Robot-Assisted Percutaneous Pedicle Screw Placement under Regional Anesthesia: A Retrospective Cohort Study

**DOI:** 10.1155/2021/6894001

**Published:** 2021-12-20

**Authors:** Shangju Gao, Jingchao Wei, Wenyi Li, Long Zhang, Can Cao, Jinshuai Zhai, Bo Gao

**Affiliations:** Department of Orthopedics, Hebei General Hospital, No. 348 Hepingxi Road, Shijiazhuang, Hebei, China

## Abstract

**Background:**

Robot-assisted pedicle screw placement is usually performed under general anesthesia to keep the body still. The aim of this study was to compare the accuracy of the robot-assisted technique under regional anesthesia with that of conventional fluoroscopy-guided percutaneous pedicle screw placement under general anesthesia in minimally invasive lumbar fusion surgery.

**Methods:**

This study recruited patients who underwent robot-assisted percutaneous endoscopic lumbar interbody fusion (PELIF) or fluoroscopy-guided minimally invasive transforaminal lumbar interbody fusion (MIS-TLIF) between December 2017 and February 2020 at a single center. Based on the method of percutaneous pedicle screw placement used, patients were divided into the robot-assisted under regional anesthesia (group RE-RO) and fluoroscopy-guided under general anesthesia (group GE-FLU) groups. The primary outcome measures were screw accuracy and the incidence of facet joint violation (FJV). Secondary outcome measures included X-ray and visual analogue scale (VAS) scores which were used to evaluate the degree of the postoperative pain at 4 hours and on postoperative days 1, 2, and 3. Intraoperative adverse events were also recorded.

**Results:**

Eighteen patients were included in group RE-RO, and 23 patients were included in group GE-FLU. The percentages of clinically acceptable screws (Gertzbein and Robbins grades A and B) were 94.4% and 91.5%, respectively. There was no significant difference in the percentages of clinically acceptable screws (*p*=0.44) or overall Gertzbein and Robbins screw accuracy grades (*p*=0.35). Only the top screws were included in the analysis of FJVs. The percentages of FJV (Babu grades 1, 2, and 3) were 5.6% and 28.3%, respectively. This difference was statistically significant (*p*=0.01). Overall, the FJV grades in group RE-RO were significantly better than those in group GE-FLU (*p*=0.009). The mean fluoroscopy time for each screw in group RE-RO was significantly shorter than that in group GE-FLU (group RE-RO: 5.4 ± 1.9 seconds and group GE-FLU: 6.8 ± 2.0 seconds; *p*=0.03). The postoperative pain between the RE-RO and GE-FLU groups was not statistically significant. The intraoperative adverse events included 1 case of registration failure and 1 case of guide-wire dislodgment in group RE-RO, as well as 2 cases of screw misplacement in group GE-FLU. No complications related to anesthesia were observed.

**Conclusion:**

Robot-assisted pedicle screw placement under regional anesthesia can be performed effectively and safely. The accuracy is comparable to the conventional technique. Moreover, this technique has the advantage of fewer FJVs and a lower radiation time.

## 1. Background

Pedicle screw fixation, a rigid surgical technique, has been widely used in spine surgery since the 1970s [1] and has been shown to stabilize the spine in a variety of spinal diseases, such as trauma, tumors, degeneration, and deformities. With the imaging guidance from fluoroscopy, freehand pedicle screw placement has been performed with high levels of accuracy. However, complications related to misplacements, such as nerve and vascular injuries, still persist. In addition, percutaneous pedicle screw implantation is associated with a high incidence of iatrogenic facet joint violation (FJV), which is an independent risk factor for adjacent segment disease (ASD) [2–4]. In addition to these patient-related disadvantages, the surgeon's intraoperative radiation exposure is becoming increasingly concerning [5–7]. Previous studies have shown that spinal surgical robots may be able to offer solutions to both of these concerns [8, 9].

Robot-assisted pedicle screw placement is usually performed under general anesthesia to keep the body still and improve screw placement accuracy. However, general anesthesia may be associated with high percentages of perioperative complications and medical costs, especially for elderly patients [10, 11]. In addition, some spine surgeons prefer patient feedback to reduce the possibility of nerve injury in some special surgeries, such as percutaneous endoscopic lumbar discectomy (PELD) and percutaneous kyphoplasty [12, 13]. Regional anesthesia has been suggested to be comfortable and safe in some open and minimally invasive spine surgeries [14].

Our medical team found that patients could remain motionless and painless during fluoroscopy-guided percutaneous pedicle screw placement under regional anesthesia in percutaneous endoscopic lumbar interbody fusion (PELIF) surgery. We predict that accurate, robot-assisted placement of pedicle screws in this patient state can be achieved. Therefore, we attempted to use a spine robot instead of fluoroscopy to guide pedicle screw placement. To the best of our knowledge, no previous study has been reported focusing on robot-assisted pedicle screw accuracy under regional anesthesia. This study, therefore, aimed to evaluate the accuracy and safety of robot-assisted pedicle screw placement under regional anesthesia in lumbar fusion surgery.

## 2. Methods

### 2.1. Patients

This study was approved by the Ethics Committee of the Hebei General Hospital before data collection and analysis. This retrospective study recruited patients with lumbar degenerative disease who underwent robot-assisted PELIF or fluoroscopy-guided minimally invasive transforaminal lumbar interbody fusion (MIS-TLIF) between December 2017 and February 2020. The diagnoses included lumbar spinal stenosis, lumbar disc herniation, and lumbar spondylolisthesis. The patients were divided into two groups according to the pedicle screw implantation method: robot-assisted under regional anesthesia (group RE-RO) and fluoroscopy-guided under general anesthesia (group GE-FLU).

The inclusion criteria were as follows: (1) scheduled 1- or 2-level PELIF or MIS-TILF surgery with either robot-assisted or fluoroscopy-guided percutaneous pedicle screw placement as the internal fixation technique; and (2) postoperative computed tomography (CT) scans taken before discharge with images meeting the measurement requirements. The exclusion criteria were as follows: (1) patients with a degree of lumbar spondylolisthesis or lumbar spondylolysis of II or higher; (2) patients with infection, tumors, or scoliosis of the spine; and (3) history of previous spinal surgery.

### 2.2. Surgical Technique

#### 2.2.1. Group RE-RO

**Figure 1:**
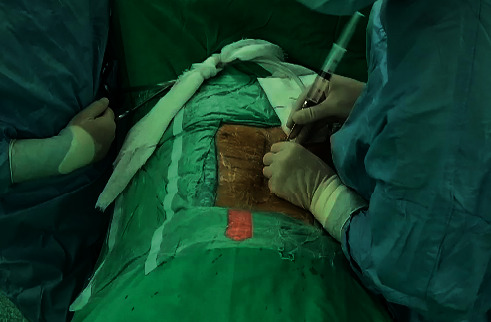
Epidural anesthesia before the operation.

All procedures were performed by the same senior spine surgeon who had performed more than 20 cases of robot-assisted surgery. The patients' CT data of the lumbar vertebrae (continuous scanning, ≤1-mm cuts) were copied from the inspection equipment and input into the robotic surgical plan workstation (Mazor Renaissance Surgical Technologies, Caesarea, Israel) for preoperative planning. Before surgery, the patient was told how to cooperate with the surgery, including keeping still and breathing evenly for some special period. During surgery, the patients were placed in a comfortable prone position on the operating table, with oxygen inhalation and ECG and vital sign monitoring. Dexmedetomidine (4 *μ*g/ml) was pumped at a rate of 3–8 ml/h. The administration of epidural anesthesia was performed using the loss-of-resistance technique through the interlaminar space of the operating segments (Figure 1). The anesthetic drug for the injection was a mixture of 0.5% lidocaine and 0.25% ropivacaine. The dose was 10 ml. In this anesthetic state, patients had hypoesthesia rather than loss of sensation in the operative region and lower extremities. The motion of the lower limbs persisted.

**Figure 2:**
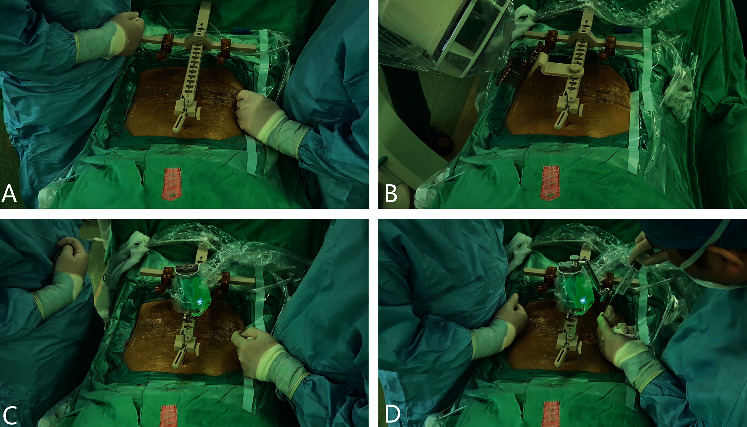
The Hover-T frame platform was fixed on the patient's spine (a); an anteroposterior image and an image 60°oblique to the plane were captured by the C-arm for registration with the preoperative CT (b); the guiding robot moved on the platform according to the preoperative plan (c); local infiltration anesthesia around the facet joints before drilling (d).

**Figure 3:**
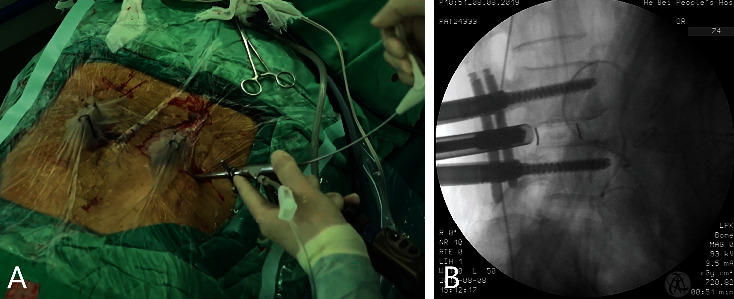
Percutaneous endoscopic lumbar interbody fusion following robot-assisted percutaneous pedicle screw implantation (a); intraoperative fluoroscopy image during the interbody fusion cage placement (b).

The surgical procedure was performed as follows: First, the working platform was installed. The Hover-T frame platform was used for all operations in this group. After local infiltration anesthesia (1% lidocaine), three needles were inserted into the spinous process of the upper lumbar spine and bilateral posterior superior iliac spines to fix the frame (Figure 2(a)). After image acquisition, registration (Figure 2(b)), and robot motion (Figure 2(c)), local infiltration anesthesia was administered to the skin and around the facet joints before incision and drilling (Figure 2(d)). To minimize deviations caused by spine movement, drilling was carried out in a painless state. Otherwise, additional local anesthesia was administered, as pedicle screw insertion could aggravate the patient's pain. It was essential to increase the speed of drug pumping in advance. Details of the robot-assisted procedure have been described in previous articles [8, 15]. After screw (minimally invasive spinal system; WEGORTHO Paedic Device Co., Ltd.; Weihai, China) placement, decompression, and interbody fusion were performed (Figure 3). No drainage system was required. Postoperative MRI and CT were necessary. Patients could walk with waist support on the first day. The protocol has been outlined in Table 1.

### 2.3. Group GE-FLU

**Figure 4:**
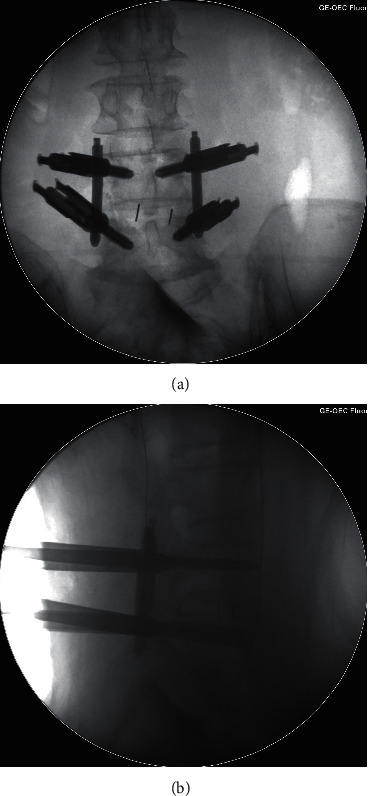
Intraoperative fluoroscopy images in group GE-FLU: AP X-ray image (a) and lateral X-ray image (b).

The pedicle screw placement procedures were completed by two senior spine surgeons who had each performed more than 50 cases of fluoroscopy-guided pedicle screw insertion. After general anesthesia, the patient was placed in a prone position. A C-arm was used to locate the targeted vertebral pedicles and plan the screw route. A puncture needle was inserted through a 1.5 cm incision with fluoroscopy guidance. After a final fluoroscopy check on the AP and lateral views, the puncture needle was replaced with a spacer. Screw (minimally invasive spinal system; WEGORTHO Paedic Device Co., Ltd.; Weihai, China) placement was performed after decompression and interbody fusion (Figure 4).

### 2.4. Outcome Evaluation

The primary outcome measures were screw accuracy and the incidence of FJV. All patients underwent thin-slice CT scans (≤1.2-mm slices) of the lumbar spine postoperatively. Screw accuracy was evaluated using the Gertzbein and Robbins criteria [16]: grade A, completely within the pedicle; grade B, < 2 mm cortical breach; grade C, 2–4 mm cortical breach; grade D, 4–6 mm cortical breach; and grade E, >6 mm cortical breach. Screw grades A and B were considered clinically acceptable [17–19]. Differences in the screw accuracy grades between the two groups and the proportions of clinically acceptable screws were assessed as the accuracy comparison parameters. FJV was evaluated only for the upper pedicle screws because of the related clinical significance using the Babu classification system [20]: grade 0, the screw does not violate the facet joint; grade 1, the screw violates the lateral facet; grade 2, the screw penetrates the articular facet by 1 mm; and grade 3, the screw lies within the articular facet surface. Differences in violation grades and the percentages of violating screws (grades 1, 2, and 3) were assessed as the FJV comparison parameters. The data were measured independently by two spinal graduate students using a picture archiving and communication system (PACS) (Neusoft Medical image diagnostic reporting system; Neusoft Co. Ltd., Shenyang, China) who were not aware of the purpose of the study in advance. If there was a discrepancy between the results, the worst result was adopted.

As secondary outcome measures, we compared the X-ray exposure and intraoperative adverse events related to the screw placement procedure as well as to anesthesia. X-ray exposure measurements were determined by the fluoroscopy time for each screw including the robot registration and intraoperative screw evaluation (sum of exposure times of the whole screw implantation and rod connecting procedures/number of screws inserted). A visual analogue scale (VAS) score was used to evaluate the degree of the postoperative pain at 4 hours and on postoperative days 1, 2, and 3.

### 2.5. Statistical Analysis

Statistical analyses were performed using IBM SPSS Statistics for Windows, version 23.0 (IBM Corp, Armonk, NY, USA). Fisher's exact test and Pearson's chi-squared test were used for group comparisons of sex, the distribution of diagnosis and screw location, as well as the percentages of clinically acceptable screws and facet violation screws. Two-sample *t* tests were used for group comparisons of age, body mass index (BMI), the superior facet joint angle, fluoroscopy time for each screw, and the VAS score at 4 hours and on postoperative days 1, 2, and 3. The Mann–Whitney *U* tests were used for group comparisons of accuracy and FJV grades. The statistical significance of these parameters was set at *p* < 0.05.

## 3. Results

Ninety-four consecutive patients were initially included in this study. Because of the requirement of postoperative CT results and other disease-related criteria, only 41 patients (22 women and 19 men) met the inclusion criteria. Eighteen patients (10 women and 8 men) who underwent PELIF were included in group RE-RO. Twenty-three patients (12 women and 11 men) were included in group GE-FLU; 20 patients (9 women and 11 men) underwent MIS-TLIF and 3 women underwent PELIF. The baseline characteristics of age, sex distribution, BMI, the superior facet joint angle, distribution of diagnosis, and screw location did not differ between the groups RE-RO and GE-FLU (Table 2).

A total of 168 screws were inserted into patients' 4 vertebrae. Among them, 74 were implanted using the robot-assisted technique under regional anesthesia (group RE-RO), and 94 were implanted using the fluoroscopy-guided technique under general anesthesia (group GE-FLU). The incidence of pedicle breach (grades B, C, D, and E) in the two groups was 10.8% (8/74) and 20.2% (19/94), respectively. There was no significant difference in the incidence of clinically acceptable screws (grades A and B), with percentages of 94.4% and 91.5% for groups RE-RO and GE-FLU, respectively (*p*=0.44). The difference in the Gertzbein and Robbins screw accuracy grades was also not statistically significant (*p*=0.35). Considering the relationship between FJV and ASD as well as the surgeon's level of concern during insertion in different segments, only the 82 top screws were included in the analysis. In group RE-RO, 5.6% of the 36 screws analyzed violated the facet joint (grades 1, 2, and 3). In group GE-FLU, the incidence of FJV was 28.3%. This difference between these two groups was statistically significant (*p*=0.01). The FJV grades in group RE-RO were significantly better than those in group GE-FLU (*p*=0.009). A detailed list of the pedicle screw accuracy grades is presented in Table 3.

The mean fluoroscopy time for each screw in group RE-RO was significantly shorter than that in group GE-FLU (group RE-RO, 5.4 ± 1.9 seconds; group GE-FLU, 6.8 ± 2.0 seconds; *p*=0.03). The VAS scores at 4 hours and on 1, 2, 3 days after surgery in group RE-RO were 4.7 ± 2.5, 4.7 ± 1.9, 3.9 ± 1.1, and 2.7 ± 1.0, which were lower than those in group GE-FLU at each time point (5.5 ± 2.0, 5.1 ± 1.5, 3.9 ± 1.3, and 2.7 ± 1.1). However, this difference was not statistically significant (*p*=0.26, *p*=0.44, *p*=0.94, and *p*=0.81). No patients suffered from neurovascular complications postoperatively or underwent revision surgery due to screw misplacement. No cases of cerebrospinal fluid (CSF) leakage or surgical site infection were observed. Other adverse events of the screw placement procedure were as follows:

1. 1 case of registration failure in group RE-RO. The screws were placed by the fluoroscopy-guided technique instead of the robot-assisted technique.
2. 1 case of guide wire dislodgment in group RE-RO. It was found during the operation, and the fragment was removed under the guidance of fluoroscopy.
3. 2 screws in 2 cases of screw misplacement in group GE-FLU. They were revised intraoperatively by the fluoroscopy-guided technique after an X-ray check. There were no complications related to anesthesia in either group.

## 4. Discussion

In this study, we showed that robot-assisted percutaneous pedicle screw placement under regional anesthesia has a high accuracy of 94.6%. The accuracy reported under general anesthesia is 85%–99% [8, 21–23]. Although the difference was not significant, group RE-RO showed higher percentages in overall grade and clinically acceptable grade compared with group GE-FLU. This outcome is clinically satisfactory. Robot-assisted screw accuracy is closely related to spine movement because of its fundamental mechanism and working principles [24]. Regional anesthesia has proved to be a safe and effective anesthetic technique under which patients can be stable and pain-free. Kang et al. reported on 111 patients who underwent open lumbar spinal decompression, endoscopic decompression, and open posterior fusion surgery under regional anesthesia [14]. The anesthetic effect was satisfactory. Xu et al. revealed an intraoperative mean VAS score of low back pain of 1.25 under epidural anesthesia during PELD surgery [12]. According to our experience, spine movement mainly affects screw accuracy by the movement of the robotic arm and screw passage drilling steps. The first step takes a short time, and cooperative patient immobility is feasible. Additional local infiltration anesthesia around the facet joints can reduce discomfort during the drilling procedure. However, once spine movement is detected, reregistration is required.

In terms of the baseline, there were some differences in the sequence of screw placement and decompression between the two groups. In the RE-RO group, to avoid the accuracy reduction in robot image acquisition and registration procedure after decompression, screw placement was carried out before decompression. In order to prevent the screw tail from affecting the decompression operation, screw placement was carried out after decompression and interbody fusion in the 20 patients who underwent MIS-TLIF. However, the screw path was established and marked with a guide wire before the decompression step. Both groups completed the screw path preparation before decompression. Therefore, we think that different decompression methods have little effect on the accuracy of screws.

In the robot-assisted cohort, the robotic platform was a Hover-T frame, which is designed for minimally invasive surgery. The frame is fixed on the spine and pelvis during the whole insertion procedure. Relative resting of the body and platform can reduce the influence of accidental body motion on screw accuracy. Ringel et al. [22] reported a lower screw accuracy of 85% by using a “bed mount” platform (a platform fixed on the edge of the operating bed) and attributed the inaccuracy to the inappropriate platform choice. The relative movement of the robot to the patient may be slightly larger with this method.

In terms of FJV events, the robot-assisted technique was better than the fluoroscopy-guided technique. This finding is consistent with previous studies [8, 25, 26]. We only analyzed the top two pedicle screws in each patient to measure the incidence of FJV for two reasons. First, only FJV from the top screws is related to ASD and even reoperation. Second, accurate measurement was difficult in the lower segments because facetectomy was performed for decompression. Furthermore, an offset from the operator might exist. To reduce the probability of FJV, surgeons should pay more attention to the entry points of the top screws during preoperative planning or intraoperative localization. Different from the robot's one-time drilling, the fluoroscopy-guided technique requires adjustment of the entry point a few times. Joint capsule injury may occur during this step. We deduce that it may aggravate the degeneration of the facet joint and cause ASD. No research has described this phenomenon.

Because of the different decompression methods, we only compared the radiation exposure time during the screw placement procedure. The results showed that the robot performed significantly better in minimizing this time. The advantage of the short radiation exposure time is more remarkable as the number of screws increases, especially in some spinal deformity surgeries. This is because most of the radiation exposure in robotic surgery occurs during the registration and platform process, which only needs to be performed once per operation. Fan et al. [18] compared the radiation dose among robots, novel guided templates, and CT-based navigation in adult degenerative scoliosis. The robot-based surgeries exhibited the lowest intraoperative radiation dose. In our study, we included the exposure time involved in connecting the percutaneous rod. The robot can preoperatively plan a better screw order, which can reduce operation times and radiation exposure. This may be one of the reasons for the low radiation exposure time.

Intraoperative adverse events were equal in the two techniques. However, it seems that the complications in the robot group are less likely to cause serious consequences. Keric et al. [27] also found no significant differences between robotic-assisted and fluoroscopy-guided screw placement regarding intraoperative complications. The identification of additional adverse events requires studies with larger sample sizes.

The findings of our study provide a new anesthesia method for the clinical application of spine robots. It proves that the spinal robot can be used under regional anesthesia. The accuracy is clinically acceptable. Considering that regional anesthesia has many advantages over general anesthesia [10], we expect that some minimally invasive operations, such as PELD, bone biopsy, and percutaneous kyphoplasty, can be performed under regional anesthesia. Medical costs and recovery periods can be reduced accordingly. There are some limitations in this study. First, this is a single-center retrospective study, so the sample size is small (only 41 patients included). Thus, selection bias may exist. Second, the preoperative and postoperative screw positions were not compared because of technical issues.

## 5. Conclusion

Robot-assisted pedicle screw placement under regional anesthesia can be performed effectively and safely. The accuracy is comparable to that of the fluoroscopy-guided technique. Moreover, this technique has the advantage of fewer FJVs and a lower radiation time.

## Figures and Tables

**Table 1:** The protocol of robot-assisted percutaneous endoscopic lumbar interbody fusion under regional anesthesia.

Key steps of this surgery
(1) Preoperative planning
(2) Patient preparation and education
(3) Monitoring and sedation
(4) Epidural anesthesia
(5) Working platform installed and robot registration
(6) Adequate local anesthesia
(7) Drilling under the guidance of the robot
(8) Pedicle screw inserted
(9) Screw evaluation with fluoroscopy
(10) Decompression and interbody fusion
(11) Postanesthetic care unit observation (1 hour)
(12) Postoperative MRI and CT (1 day after surgery)
(13) Walking with waist support (1 day after surgery)

**Table 2:** Baseline characteristics.

Characteristics	Group EP-RO	Group GE-FLU	Overall	*p* value
No. of patients ^*∗*^	18	23	41	
Female sex (%) ^*∗*^	55.6	52.2	53.7	0.83
Age (years)^✟^	61.6 ± 7.1	62.4 ± 6.1	62.1 ± 6.5	0.71
Mean BMI (kg/m^2^)^✟^	26.0 ± 3.6	25.5 ± 2.9	25.7 ± 3.2	0.58
Superior facet joint angle^✟^	44.1 ± 3.6	43.8 ± 2.1	44.0 ± 3.2	0.56
Diagnoses ^*∗*^				0.88
LDH	4	5	9	
LSS	9	10	19	
Lumbar spondylolisthesis	5	8	13	
Location of screws ^*∗*^				0.98
L3	3	4	7	
L4	14	19	33	
L5	16	20	36	
S1	4	4	8	

^*∗*^Values are the number or the number (%) of patients. ^✟^Values are presented as mean ± SD. BMI indicates body mass index.

**Table 3:** Comparison of pedicle screw placement accuracy, FJV, and fluoroscopy time.

Characteristics	Group EP-RO	Group GE-FLU	Total	*p*value
No. of screws ^*∗*^	74	94	168	
Accuracy grade ^*∗*^				
(*n*%)				0.102
Grade A	66 (89.2%)	75 (79.8%)	141 (83.9%)	
Grade B	4 (5.4%)	11 (11.7%)	15 (8.9%)	
Grade C	4 (5.4%)	5 (5.3%)	9 (5.4%)	
Grade D	—	2 (2.1%)	2 (1.2%)	
Grade E	—	1 (1.1%)	1 (0.6%)	
Clinically acceptable ^*∗*^				
(Grade A + B)	70 (94.6%)	86 (91.5%)	156 (92.8%)	0.44
No. of screws for FJV comparison ^*∗*^	36	46	82	
FJV grade ^*∗*^				
(*n*%)				0.009
Grade 0	34 (94.4%)	33 (71.7%)	67 (81.7%)	
Grade 1	1 (2.8%)	8 (17.4%)	9 (11.0%)	
Grade 2	1 (2.8%)	2 (4.3%)	3 (3.7%)	
Grade 3	—	3 (6.5%)	3 (3.7%)	
Violating screws^*∗*^				
(Grade 1 + 2+3)	2(5.6%)	13(28.3%)	15(18.3%)	0.01
Fluoroscopy time per screw^✟^ (secs)	5.4 ± 1.9	6.8 ± 2.0	6.2 ± 2.0	0.03
Postoperative pain rating ^✟^ (VAS)				
4 hours after surgery	4.7 ± 2.5	5.5 ± 2.0		0.26
Postoperative day 1	4.7 ± 1.9	5.1 ± 1.5		0.44
Postoperative day 2	3.9 ± 1.1	3.9 ± 1.3		0.94
Postoperative day 3	2.7 ± 1.0	2.7 ± 1.1		0.81

^*∗*^Values are the number or the number (%) of patients. ^✟^Values are presented as the mean ± SD. FJV indicates facet joint violation. VAS indicates visual analogue score.

## Data Availability

The data are available upon request to the corresponding author.

## Abbreviations

PELIF: Percutaneous endoscopic lumbar interbody fusion
MIS-TLIF: Minimally invasive transforaminal lumbar interbody fusion
RE-RO: Robot-assisted under regional anesthesia
GE-FLU: Fluoroscopy-guided under general anesthesia
FJV: Facet joint violation
ASD: Adjacent segment disease
PELD: Percutaneous endoscopic lumbar discectomy
VAS: Visual analogue scale
CSF: Cerebrospinal fluid.
